# *In Vitro* Antibacterial Activity of Rhodanine Derivatives against Pathogenic Clinical Isolates

**DOI:** 10.1371/journal.pone.0164227

**Published:** 2016-10-06

**Authors:** Ahmed AbdelKhalek, Charles R. Ashby, Bhargav A. Patel, Tanaji T. Talele, Mohamed N. Seleem

**Affiliations:** 1 Department of Comparative Pathology, College of Veterinary Medicine, Purdue University, 625 Harrison Street, West Lafayette, Indiana, 47907, United States of America; 2 Department of Pharmaceutical Sciences, College of Pharmacy and Health Sciences, St. John’s University, 8000 Utopia Parkway, Queens, New York, 11439, United States of America; 3 Purdue Institute for Inflammation, Immunology, and Infectious Disease, Purdue University, West Lafayette, Indiana, 47907, United States of America; Cornell University, UNITED STATES

## Abstract

Bacterial infections present a serious challenge to healthcare practitioners due to the emergence of resistance to numerous conventional antibacterial drugs. Therefore, new bacterial targets and new antimicrobials are unmet medical needs. Rhodanine derivatives have been shown to possess potent antimicrobial activity via a novel mechanism. However, their potential use as antibacterials has not been fully examined. In this study, we determined the spectrum of activity of seven rhodanine derivatives (compounds Rh **1**–**7**) against clinical isolates of Gram-positive and Gram-negative bacterial strains and *Candida albicans*. We also synthesized and tested three additional compounds, ethyl ester and amide of rhodanine **2** (Rh **8** and Rh **10**, respectively) and ethyl ester of rhodanine **3** (Rh **9**) to determine the significance of the carboxyl group modification towards antibacterial activity and human serum albumin binding. A broth microdilution assay confirmed Rh **1**–**7** exhibit bactericidal activity against Gram-positive pathogens. Rh **2** had significant activity against various vancomycin-resistant (MIC_90_ = 4 μM) and methicillin-resistant (MIC_90_ = 4 μM) *Staphylococcus aureus* (VRSA and MRSA), *Staphylococcus epidermidis* (MIC = 4 μM) and vancomycin-resistant *Enterococcus* (VRE) *strains* (MIC_90_ = 8 μM). The rhodanine compounds exhibited potent activity against *Bacillus* spp., including *Bacillus anthracis*, with MIC range of 2–8 μM. In addition, they had potent activity against *Clostridium difficile*. The most potent compound, Rh **2**, at 4 and 8 times its MIC, significantly decreased *S*. *epidermidis* biofilm mass by more than 35% and 45%, respectively. None of the rhodanine compounds showed antimicrobial activity (MIC > 128 μM) against various 1) Gram-negative pathogens (*Acinetobacter baumannii*, *Escherichia coli*, *Klebsiella pneumonia*, *Pseudomonas aeruginosa*, and *Salmonella Typhimurium*) or 2) strains of *Candida albicans* (MIC > 64 μM). The MTS assay confirmed that rhodanines were not toxic to mouse murine macrophage (J774.1A) up to 64 μM, human keratinocytes (HaCat) up to 32 μM, and human ileocecal colorectal cell (HRT-18) up to 128 μM. Overall, these data suggest that certain rhodanine compounds may have potential use for the treatment of several multidrug-resistant Gram-positive bacterial infections.

## Introduction

Infections caused by multidrug-resistant Gram-positive and Gram-negative bacteria have become a major problem, particularly in hospitalized patients. For example, there are now strains of multidrug resistant *Staphylococcus aureus* and *Enterococci* that have become resistant to last-resort drugs. In addition, various Gram-negative bacteria, including *Pseudomonas aeruginosa*, *Acinetobacter baumannii*, certain *Escherichia coli* and *Klebsiella pneumoniae* strains have acquired genes that produce multidrug resistance.

One potential way to surmount resistance is to synthesize compounds that are structurally distinct from the currently approved antibiotics. Previously, we reported that certain rhodanine derivatives had bactericidal activity *in vitro* (three compounds with MIC = 0.98–1.95 μg/mL and six compounds with MIC = 1.95–3.90 μg/mL) against methicillin-resistant *Staphylococcus aureus* (MRSA) strains from different body areas and global locations [1]. In addition, a number of the rhodanines were highly active against a multidrug-resistant strains of MRSA (MRSA ATCC BAA39 which is resistant to at least 9 different antibacterial drugs and MRSA ATCC 700698 which has reduced susceptibility to vancomycin) [1]. Previous structure-activity relationship studies of this class of rhodanine compounds suggested the important role of 1) a hydrophobic aromatic group at the 3-position of the benzylidene moiety, 2) the type and nature of connecting group between the two aromatic rings of the benzylidene moiety and 3) stereochemical configuration at the phenylalanine segment [1]. Subsequently, we showed that the active rhodanine compounds were producing their antibacterial activity by inhibition of DNA gyrase and topoisomerase IV via a novel mechanism [2]. However, the effect of our rhodanine compounds against other strains of MRSA, as well as other Gram-positive and Gram-negative bacteria and fungi, remained to be determined. Therefore, in this study, we selected seven representative rhodanine derivatives for extensive antimicrobial evaluation.

**Fig 1:**
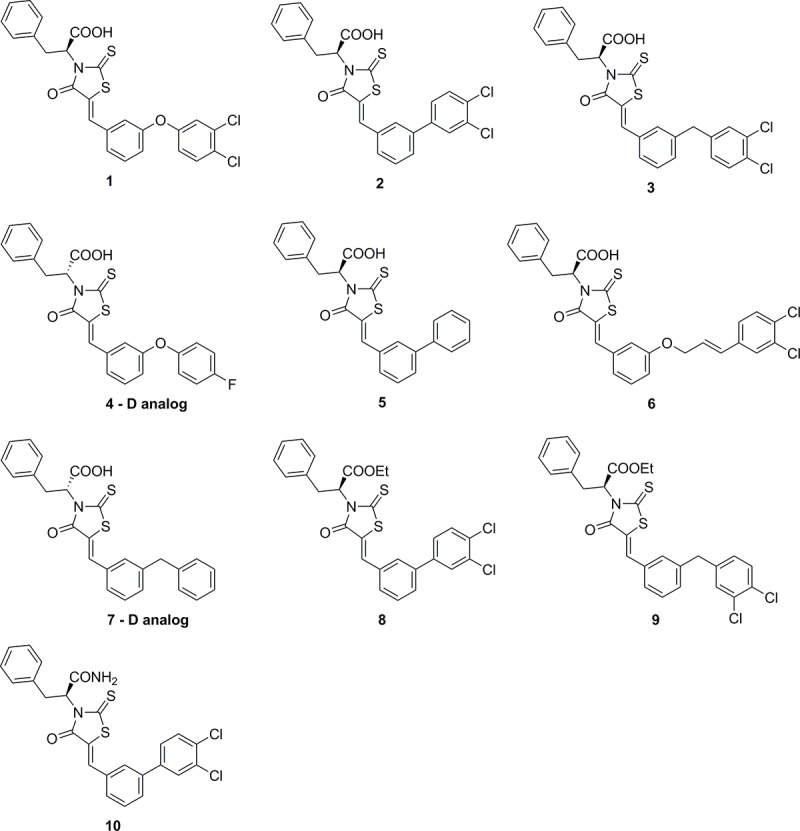
Chemical structures of rhodanine compounds 1–10 utilized in this study.

The goal of this study was to determine the *in vitro* antimicrobial activity of seven rhodanine derivatives **1**–**7 (Fig 1)** against a wider panel of Gram-positive and Gram-negative bacterial strains as well as *Candida albicans*. In addition, we wanted to assess whether these compounds had efficacy against staphylococcal biofilms using an *in vitro* model of *S*. *epidermidis*. Furthermore, we assessed the toxicity of rhodanines against three cell lines that represent three routes of administration (systemic, topical and oral).

## Materials and Methods

### Synthesis of compounds 1–10

The syntheses of compounds **1** and **4**–**6** [3] and **2**, **3** and **7** [1] was previously reported by our group and that for compounds **8**–**10** is reported herein (see Supporting Information). LogP and LogS were predicted for all the compounds to assess their lipophilicity and water solubility using QikProp, version 4.8, Schrödinger, LLC, NY.

### Bacterial strains and reagents

The bacterial strains used in this study are presented in S1 Table. Murine macrophage (J774A.1), human keratinocytes (HaCat) and human ileocecal colorectal (HRT-18) cell lines were purchased from ATCC (Manassas, VA), vancomycin hydrochloride (Gold Biotechnology, St. Louis, MO, USA), linezolid, amphotericin B, fusidic acid (Chem-impex International, Wood Dale, IL, USA), ciprofloxacin, gentamycin (Enzo Life Sciences, Farmingdale, NY, USA), rifampicin, erythromycin (Sigma-Aldrich, St. Louis, MO, USA), oxacillin (TCI chemicals, Portland, OR, USA), fluconazole (Acros, NJ, USA), Daptomycin (Selleckchem, Houston, TX, USA) and colistin (Alfa Aesar, Ward Hill, MA, USA) were acquired from other commercial vendors. Trypticase soy agar (TSA), Trypticase soy broth (TSB), Brain heart infusion, Yeast peptone dextrose (YPD) agar and broth and Anaerobic blood agar as well as the Anaerobic gas pack system were purchased from Becton, Dickinson and Company (Cockeysville, MD). Phosphate buffered saline (corning), DMEM, Agar, glucose and crystal violet (Sigma-Aldrich), Middlebrook 7H9 broth base and the supplementary OADC vials (HiMedia Laboratories, PA, USA), Middlebrook 7H11 agar base (CRITERION, Santa Maria, CA, USA), Fetal bovine serum (ATCC), and MTS (Promega, Madison, WI, USA) were also used in the study. For *Clostridium* work, Brain heart infusion medium was supplemented with yeast extract, L-cysteine, Vitamin K1 and Hemin (Sigma-Aldrich, St. Louis, MO, USA).

### The minimum inhibitory concentration (MIC) and the minimum bactericidal concentration (MBC) of Rhodanine derivatives

The minimum inhibitory concentration (MIC) and the minimum bactericidal concentration (MBC) of rhodanine compounds **1** to **7** were determined against various Gram-positive and Gram-negative pathogens (S1 Table) following the guidelines of the Clinical and Laboratory Standards Institute (CLSI)[4]. The broth microdilution technique was used, followed by subculturing on agar plates that were rhodanine—free. Bacteria (~1.5x10^5^ CFU/mL) and the test compounds (1–128 μM) were placed together in a 96 well-plate and incubated at 37°C for 24 hours and the agar plates were incubated at 37°C for 24 hours. The MICs reported represent the lowest concentration of each compound necessary to inhibit bacterial growth and the MBCs represent the lowest concentration required to reduce the initial bacterial inoculum by ≥ 99.9%.

### MICs of the rhodanine compounds against *Clostridium difficile*

Clinical isolates of *C*. *difficile* were cultured on anaerobic blood agar and incubated anaerobically using container gas pack system at 37° C for 48 hours. The colonies were then suspended in pre-reduced phosphate buffered saline (PBS) and adjusted to 0.5 McFarland standard then diluted 1:300 in pre-reduced Supplemented Brain Heart Infusion broth. The bacterial suspension was then transferred to each well of 96-well plates, the drugs were added to the first row of wells in the required concentrations and serially diluted along the plates. The plates were incubated again anaerobically using container gas pack system at 37° C for 48 hours. The (MIC) recorded was the lowest concentration of the drug showing no visible growth of the bacteria.

### Biofilm eradication activity of Rhodanine compounds

We evaluated the efficacy of the most potent compound (rhodanine **2)** to disrupt established biofilms produced by methicillin-resistant *Staphylococcus epidermidis* (MRSE) using the microtiter dish biofilm formation assay [5–9]. We used *S*. *epidermidis* ATCC 35984 (NRS 101), a high-slime producer isolated in septicemic patients, with colonized intravascular catheters from Tennessee, USA [10]. This strain is a multi-drug resistant strain, showing resistance to methicillin, erythromycin, kanamycin, gentamicin, clindamycin and trimethoprim [10]. Briefly, an overnight culture of biofilm—producing MRSE was diluted 1:100 in a fresh medium containing 1% glucose in a 96-well tissue-culture treated plate. Bacteria were incubated at 37°C for 24 h to permit the formation of an adherent biofilm. The medium was removed and the biofilm was washed with PBS. Antibacterial drugs (vancomycin and linezolid) and rhodanine **2**, at indicated concentration, were added and incubated again at 37°C for 24 h. Plates were washed again and biofilms were stained with 0.1% (wt/vol) crystal violet. Plates were washed with PBS, air-dried and biofilm mass was dissolved using 95% ethanol. The intensity of crystal violet was measured using a micro plate reader (SpectraMax i3x; Molecular Devices, Sunnyvale, CA, USA). Data are presented as the percent biofilm mass reduction in treated groups in relation to untreated wells.

### The cytotoxicity of Rhodanine compounds against a murine macrophage (J774.A1) and human keratinocytes (HaCat) cell lines

Rhodanine compounds were assayed at concentrations of 16 μM, 32 μM, 64 μM, and 128 μM against a murine macrophage cell line (J774.A1) and human keratinocyte cell line (HaCat) to determine the potential toxic effect in mammalian cells [11]. Briefly, ~2 x 10^4^ cells /well suspended in 200 μL of DMEM supplemented with 10% fetal bovine serum (FBS), L-glutamine, NaHCO_3_, pyridoxine-HCl, and 45,000 mg/L glucose were seeded in 96-well plates and incubated at 37°C in a 5% CO_2_ atmosphere. The cells were cultured for 48 hours (60% confluency) before the assays. The cells were further incubated with 16 μM, 32 μM, 64 μM, and 128 μM of rhodanine compounds for 2 hours. The culture media were discarded, and the cells in each well were washed with PBS and 100 μL of cell culture media were added prior to addition of the assay reagent MTS3-(4,5-dimethylthiazol-2-yl)-5-(3-carboxymethoxyphenyl)-2-(4-sulfophenyl)-*2H*-tetrazolium) (Promega, Madison, WI, USA). The plates were incubated for 4 hours at 37°C in a humidified 5% CO_2_ atmosphere. The absorbance at 490 nm was recorded and corrected absorbance readings (actual absorbance readings for each treatment subtracted from background absorbance) were taken using a kinetic ELISA microplate reader (SpectraMax i3x, Molecular Devices, Sunnyvale, CA, USA). The quantity of viable cells after treatment with each compound was expressed as a percentage of the control, DMSO.

### Cytotoxicity of Rhodanine compounds against human ileocecal colorectal cell line (HRT-18)

Rhodanine compounds were assayed at concentrations of 32 μM, 64 μM, 128 μM, and 256 μM against a human ileocecal colorectal cell line (HRT-18) to determine the potential toxic effect in intestinal mammalian cells. Briefly, ~2 x 10^4^ cells suspended in 100 μL of RPMI-1640 supplemented with 10% horse serum were seeded in a 96-well plate and incubated at 37°C in a 5% CO_2_ atmosphere. The cells were cultured for 24 hours (90% confluency) before the assays. The cells were further treated as above.

### Antimicrobial activity of rhodanine compounds in the presence of human serum albumin

The antimicrobial activity of rhodanine compounds in the presence of 4% human serum albumin (HSA) was tested against MRSA USA300. The MICs of rhodanine compounds and control antibiotics (vancomycin and daptomycin) were tested as described in the methods above using tryptic soy broth spiked with 4% HSA. We also synthesized and tested three more compounds, ethyl ester and amide of rhodanine **2** (Rh **8** and Rh **10**, respectively) and ethyl ester of rhodanine **3** (Rh **9**) to determine the influence of a carboxyl group modification toward anti-MRSA activity and human serum albumin (HSA) binding.

### The effect of outer membrane and efflux pump of Gram-negative bacteria on rhodanines resistance

The MIC of the rhodanines and control antibiotics, in the presence of a sub-inhibitory concentration of colistin or polymixin B nonapeptide (PMBN), against Gram-negative bacteria was evaluated as described before [7,8]. The antibacterial activity of the rhodanines was further investigated against *E*. *coli* SM1411Δ acrAB, a strain that is deficient in the multidrug-resistant AcrAB efflux pump, as described before [7,8].

## Results

### Lipophilicity of rhodanine compounds 1 to 7

Calculated log P and log S (clog P and clog S) values were used to assess the lipophilicity of rhodanine compounds (Table 1). All of the rhodanine compounds exhibited clog P value of >5 and a clog S value of < -5, which indicates that these compounds are highly lipophilic and predicted to bind to plasma proteins[12].

**Table 1:** Solubility predictors (clog P and clog S) of rhodanine compounds.

Compound	clog P	clog S
**Rh 1**	7.405	-8.555
**Rh 2**	7.586	-8.833
**Rh 3**	7.895	-9.209
**Rh 4**	6.258	-5.336
**Rh 5**	6.408	-7.354
**Rh 6**	7.627	-8.138
**Rh 7**	6.81	-7.271
**Rh 8**	7.69	-8.066
**Rh 9**	8.082	-8.852
**Rh 10**	5.981	-7.583

### *In vitro* antibacterial activity of rhodanine compounds 1 to 7 against Gram-positive cocci (VRE, MRSA, and VRSA)

The *in vitro* activity of rhodanine compounds **1**–**7** was determined initially against vancomycin-resistant *Enterococci* (VRE), methicillin-resistant *Staphylococcus aureus* (MRSA), and vancomycin-resistant *Staphylococcus aureus* (VRSA) as shown in Tables 2–4. The rhodanine compounds exhibited potent bactericidal activity against all tested bacteria including strains that are resistant to conventional antimicrobials such as vancomycin and linezolid (Tables 2–4). The minimum inhibitory concentration (MIC) of rhodanine required to inhibit 50% (MIC_50_) and 90% (MIC_90_) of VRE, MRSA, and VRSA ranged from 4 μM to 32 μM. The rhodanine compounds retained their antibacterial activity against an array of bacterial strains (VRE, MRSA, and VRSA) exhibiting resistance to numerous antibiotic classes including glycopeptides, oxazolidones, tetracycline, β-lactams, macrolides, and aminoglycosides.

**Table 2:** Minimum inhibitory concentration (MIC) and minimum bactericidal concentration (MBC) of rhodanine compounds (μM) against vancomycin resistant *enterococci* (VRE).

VRE Strains	MIC/MBC μM								
	Rh 1	Rh 2	Rh 3	Rh 4	Rh 5	Rh 6	Rh 7	Vancomycin	Linezolid
***E*. *faecalis* R712 HM-335**	4/8	4/4	4/4	16/32	8/16	4/8	8/16	> 64/—	2/32
***E*. *faecalis* ERV103 HM-934**	8/8	4/4	4/8	32/32	16/16	8/16	16/16	> 64/—	2/>64
***E*. *faecalis* S613 HM-334**	8/8	4/4	4/8	32/32	16/16	8/16	16/16	> 64/—	2/64
***E*. *faecalis* TX0104 HM-201**	8/8	4/4	4/8	32/32	16/16	8/8	16/16	> 64/—	2/>64
***E*. *faecalis* NR31972 Strain SF 28073**	8/32	4/32	4/64	32/32	16/16	8/16	16/16	> 64/—	2/32
***E*. *faecium* NR31914 Strain E0120**	8/64	4/32	8/64	32/64	16/>64	8/64	16/32	> 64/—	2/>64
***E*. *faecium* Patient #1–1 NR-31903**	4/16	4/64	4/64	16/64	8/64	4/>64	8/16	> 64/—	16/>64
***E*. *faecium* E417 HM-965**	8/>64	4/64	4/64	16/>64	8/>64	4/>64	8/64	> 64/—	2/>64
***E*. *faecium* E1071 NR-28978**	8/64	8/64	8/>64	32/64	16/>64	8/>64	16/>64	> 64/—	2/>64
***E*. *faecium* HM 968 Strain ERV102**	8/32	4/64	4/64	32/64	16/64	8/>64	16/>64	> 64/—	2/>64
**MIC 50**	8	4	4	32	16	8	16	>64	2
**MIC 90**	8	4	8	32	16	8	16	>64	2

**Table 3:** Minimum inhibitory concentration (MIC) and minimum bactericidal concentration (MBC) of rhodanine compounds (μM) against methicillin-resistant *Staphylococcus aureus* (MRSA).

MRSA strains NRS number	MIC/MBC μM						
	Rh 1	Rh 2	Rh 3	Rh 4	Rh 5	Rh 6	Rh 7
**384**	8/32	4/16	16/16	16/64	8/64	4/4	8/64
**107**	8/8	8/16	8/32	16/32	16/16	8/8	16/16
**385**	8/8	4/4	16/16	16/32	8/16	4/4	8/16
**386**	8/16	4/8	8/32	16/>64	8/64	4/32	8/>64
**383**	8/8	4/4	8/8	32/32	16/16	4/8	16/16
**19**	8/8	4/4	8/8	16/32	8/16	8/8	8/8
**1**	8/8	4/4	8/8	16/32	8/16	8/8	8/16
**382**	8/8	4/4	16/32	16/16	8/8	4/16	16/1
**37**	8/8	4/4	8/16	32/32	16/16	8/16	16/16
**119**	8/8	4/4	8/8	16/32	8/8	4/4	8/8
**387**	8/8	4/4	16/32	16/64	8/8	4/8	8/8
**MIC 50**	8	4	8	16	8	4	8
**MIC 90**	8	4	16	32	16	8	16

**Table 4:** Minimum Inhibitory Concentration (MIC) and minimum bactericidal concentration (MBC) of rhodanine compounds (μM) against Vancomycin Resistant *Staphylococcus aureus* (VRSA) strains.

VRSA strains	MIC/MBC μM							
	Rh 1	Rh 2	Rh 3	Rh 4	Rh 5	Rh 6	Rh 7	Vancomycin
**VRSA 13**	8/32	4/64	8/32	16/64	8/16	8/32	8/32	> 64/—
**VRSA 12**	8/32	4/32	8/32	16/64	8/16	4/32	8/16	> 64/—
**VRSA 11b**	8/32	4/64	8/16	16/64	8/16	4/32	8/32	> 64/—
**VRSA 11a**	8/16	4/4	8/16	16/64	8/16	4/64	8/16	> 64/—
**VRSA 10**	8/32	4/32	8/64	16/32	8/16	4/16	8/16	> 64/—
**VRSA 3a**	8/16	4/16	16/16	16/32	8/16	8/8	8/8	> 16
**VRSA 2**	8/8	4/4	8/16	16/16	8/8	8/8	8/8	> 16
**VRSA 3b**	8/8	4/4	8/8	16/16	8/16	8/16	8/32	> 16
**VRSA 4**	8/8	4/4	8/32	16/64	8/16	4/8	8/16	> 64/—
**VRSA 5**	8/16	4/64	8/64	16/32	8/16	4/16	8/8	64/>64
**VRSA 1**	8/8	4/8	8/32	16/64	8/16	8/> 64	16/32	> 64/—
**VRSA 6**	4/8	4/16	8/16	16/32	8/16	4/32	8/16	> 64/—
**VRSA 7**	8/8	4/32	8/32	16/32	8/32	8/8	8/16	> 64/—
**VRSA 8**	4/32	4/8	8/16	16/32	8/16	4/16	8/16	> 64/—
**VRSA 9**	8/32	8/32	8/16	32/32	16/16	8/16	16/16	> 64/—
**MIC 50**	8	4	8	16	8	4	8	>64
**MIC 90**	8	4	8	16	8	8	16	>64

### *In vitro* antibacterial activity of rhodanine compounds 1 to 7 against *Bacillus anthracis*

The rhodanine compounds exhibited MIC and MBC values of 2–4 μM against *B*. *anthracis* strains, comparable to the MIC and MBC values of vancomycin and linezolid as shown in Table 5. However, they are less efficacious compared to the ciprofloxacin. Furthermore, the rhodanine compounds retained their antibacterial activity against ciprofloxacin-resistant *B*. *anthracis*. Interestingly, the MIC and MBC values are the same for all of the tested rhodanine compounds.

**Table 5:** Minimum Inhibitory Concentration (MIC) and minimum bactericidal concentration (MBC) of rhodanine compounds against *Bacillus anthracis* (Anthrax).

*Bacillus anthracis* strains	MIC/MBC μM								
	Rh 1	Rh 2	Rh 3	Rh 4	Rh 5	Rh 6	Rh 7	Ciprofloxacin	Gentamicin
***Bacillus anthracis* AMES35**	2/2	2/2	2/2	4/4	2/2	2/2	2/2	0.125/0.125	0.25/0.25
***Bacillus anthracis* UM23**	2/2	2/2	2/2	4/4	2/2	2/2	2/2	< 0.0625/< 0.0625	0.25/0.25
***Bacillus anthracis* Weybridge**	2/2	2/2	2/2	4/4	2/4	2/2	2/2	>128/—	< 0.0625/< 0.0625

### *In vitro* antibacterial activity of rhodanine compounds 1 to 7 against *Bacillus* strains

In general, rhodanine compounds **1**–**7**, with the exception of compound **4**, showed MIC values ranging from 2 μM to 8 μM against 10 different *Bacillus* strains (Table 6). Interestingly, the MBC values are the same or few fold higher as that of MIC values.

**Table 6:** Minimum inhibitory concentration (MIC) and minimum bactericidal concentration (MBC) of rhodanine compounds (μM) against *Bacillus* Strains.

*Bacillus*Strains	MIC/MBC μM									
	Rh 1	Rh 2	Rh 3	Rh 4	Rh 5	Rh 6	Rh 7	Vancomycin	Linezolid	Ciprofloxacin
***B*. *cereus* VD148 NR-22150**	4/4	2/2	8/8	8/8	4/4	4/4	4/4	< 0.5/< 0.5	2/2	< 0.25/< 0.25
***B*. *licheniformis* NRS 712 NR-2499**	4/4	4/4	4/4	16/16	8/8	4/4	8/8	< 0.5/< 0.5	2/4	< 0.25/< 0.25
***B*. *licheniformis* Gibson 46 (NCIB 9375) NR-2494**	2/2	2/2	2/2	16/16	8/8	4/4	8/8	< 0.5/2	1/2	< 0.25/< 0.25
***B*. *cereus* VD115 NR-22148**	4/4	4/4	4/4	8/8	4/4	4/4	4/4	< 0.5/< 0.5	< 0.5/1	< 0.25/< 0.25
***B*. *cereus* BAG1X1-1 NR-28575**	2/2	2/2	2/2	8/8	4/4	4/4	2/4	< 0.5/< 0.5	< 0.5/1	< 0.25/< 0.25
***B*. *cereus* BAG1O-2 NR-28582**	4/4	4/4	4/4	8/8	4/4	4/4	4/4	< 0.5/< 0.5	2/2	< 0.25/< 0.25
***B*. *cereus* BAG1X2-1 NR-28578**	4/4	2/4	4/4	8/8	4/4	4/4	4/4	< 0.5/< 0.5	1/2	< 0.25/< 0.25
***B*. *cereus* NRS 201 NR-2488**	4/4	4/4	4/4	8/> 64	4/4	4/4	4/4	< 0.5/< 0.5	2/4	< 0.25/< 0.25
***B*. *cereus* G9241 NR-9564**	4/4	4/4	4/4	16/16	8/8	4/4	8/8	< 0.5/< 0.5	1/1	< 0.25/< 0.25
***B*. *cereus* VD014 NR-22141**	4/4	2/2	4/4	8/8	4/8	4/4	4/4	< 0.5/< 0.5	2/4	< 0.25/< 0.25
**MIC 50**	4	2	4	8	4	4	4	< 0.5	1	< 0.25
**MIC 90**	4	4	4	16	8	4	8	< 0.5	2	< 0.25

### *In vitro* antibacterial activity of rhodanine compounds 1 to 7 against *Clostridium difficile*

Rhodanine compounds **1**–**7** showed MIC values ranging from 1 μM to 8 μM against five strains of *C*. *difficile* (Table 7).

**Table 7:** Minimum inhibitory concentration (MIC) of rhodanine compounds (μM) against *Clostridium difficile*.

*C*. *difficile* Strains	MIC μM								
	Rh 1	Rh 2	Rh 3	Rh 4	Rh 5	Rh 6	Rh 7	Vancomycin	Metronidazole
**HM-746**	4	2	2	2	2	2	2	0.25	0.125
**HM-88**	4	4	2	4	4	4	4	0.5	0.25
**Isolate-1 NR-13427**	4	2	2	2	4	4	4	1	0.25
**Toxigenic Strain P8 NR-32888**	4	8	4	4	4	8	4	0.5	1
**HM-745**	2	2	1	2	4	4	1	0.125	0.5

### *In vitro* antibacterial activity of rhodanine compounds 1 to 7 against *Mycobacterium smegmatis*

Rhodanine compounds **1**–**3** showed MIC value of 4 μM for *M*. *smegmatis*. Rhodanine compounds **4**–**7** were less potent showing MICs value of 16–32 μM (Table 8).

**Table 8:** Minimum inhibitory concentration (MIC) and minimum bactericidal concentration (MBC) of rhodanine compounds (μM) against *Mycobacterium smegmatis*.

	MIC/MBC μM									
	Rh 1	Rh 2	Rh 3	Rh 4	Rh 5	Rh 6	Rh 7	Vancomycin	Linezolid	Rifampicin
***M*. *Smegmatis* ATCC 14468**	4/8	4/8	4/8	32/>64	16/16	16/32	16/16	2/16	4/8	32/>64

### *In vitro* antibacterial activity of rhodanine compounds 1 to 7 against Gram-negative bacteria and *Candida albicans*

None of the rhodanine compounds showed activity against Gram-negative bacteria (*P*. *aeruginosa*, *K*. *pneumoniae*, *Acinetobacter spp*., *Salmonella typhimurium and E*. *coli*) or *C*. *albicans* at the 128 μM, the highest tested concentration (Tables 9 and 10).

**Table 9:** Minimum Inhibitory Concentration (MIC) of rhodanine compounds Gram-negative pathogens.

Bacterial strain	MIC μM							
	Rh 1	Rh 2	Rh 3	Rh 4	Rh 5	Rh 6	Rh 7	Gentamicin
***P*. *aeruginosa* ATCC 15442**	>128	>128	>128	>128	>128	>128	>128	1
***P*. *aeruginosa* ATCC 9721**	>128	>128	>128	>128	>128	>128	>128	0.5
***K*. *pneumoniae* NR-15412**	>128	>128	>128	>128	>128	>128	>128	8
***K*. *pneumoniae* NR-15417**	>128	>128	>128	>128	>128	>128	>128	32
***Acinetobacter baumannii* ATCC 13345**	>128	>128	>128	>128	>128	>128	>128	16
***Acinetobacter baumannii* ATCC 17786**	>128	>128	>128	>128	>128	>128	>128	128

**Table 10:** Minimum inhibitory concentration (MIC) of rhodanine compounds against *Candida albicans*.

*Candida* Strains	MIC								
	Rh 1	Rh 2	Rh 3	Rh 4	Rh 5	Rh 6	Rh 7	Fluconazole	Amphotericin B
***C*. *albicans* NR 29435**	>64	>64	>64	>64	>64	>64	>64	< 0.5/>64	1/2
***C*. *albicans* ATCC 10231**	>64	>64	>64	>64	>64	>64	>64	< 0.5/1	1/1
***C*. *albicans* NR 294436**	>64	>64	>64	>64	>64	>64	>64	< 0.5/>64	2/2
***C*. *albicans* NR 29449**	>64	>64	>64	>64	>64	>64	>64	< 0.5/>64	1/2
***C*. *albicans* NR29438**	>64	>64	>64	>64	>64	>64	>64	< 0.5/>64	1/4
***C*. *albicans* NR 29434**	>64	>64	>64	>64	>64	>64	>64	< 0.5/1	2/2
***C*. *albicans* NR29437**	>64/—	>64/—	>64/—	>64/—	>64/—	>64/—	>64/—	1/ND	2/ND
***C*. *albicans* NR 29453**	>64/—	>64/—	>64/—	>64/—	>64/—	>64/—	>64/—	< 0.5/< 0.5	1/2
***C*. *albicans* NR 29448**	>64/—	>64/—	>64/—	>64/—	>64/—	>64/—	>64/—	> 64/—	2/2
***C*. *albicans* NR 29446**	>64/—	>64/—	64/—	64/—	>64/—	>64/—	64/—	> 64/—	1/1

### Anti-biofilm activity of rhodanine 2 against *Staphylococcus epidermidis*

**Fig 2:**
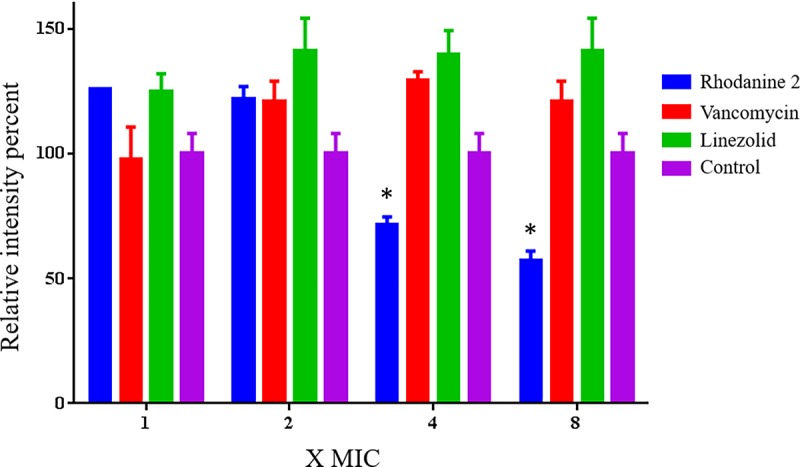
Efficacy of rhodanine compounds 2, vancomycin and linezolid (all at 1, 2, 4, and 8 X MIC μM) in disrupting an established methicillin-resistant *S*. *epidermidis* biofilm.

To determine the efficacy of the rhodanine compounds to mitigate the impact of Staphylococcal biofilms, we investigated the effect of rhodanine **2** on pre-formed methicillin-resistant *S*. *epidermidis* biofilms as shown in Fig 2. Rhodanine **2**, at 4 and 8 times its MIC, significantly reduced *S*. *epidermidis* biofilm mass by more than 35% and 45%, respectively. In contrast, even at high concentrations, neither linezolid nor vancomycin significantly reduce biofilm formation.

### Cytotoxicity of rhodanine compounds against J774A.1, HaCat and HRT-18 cell lines

**Fig 3:**
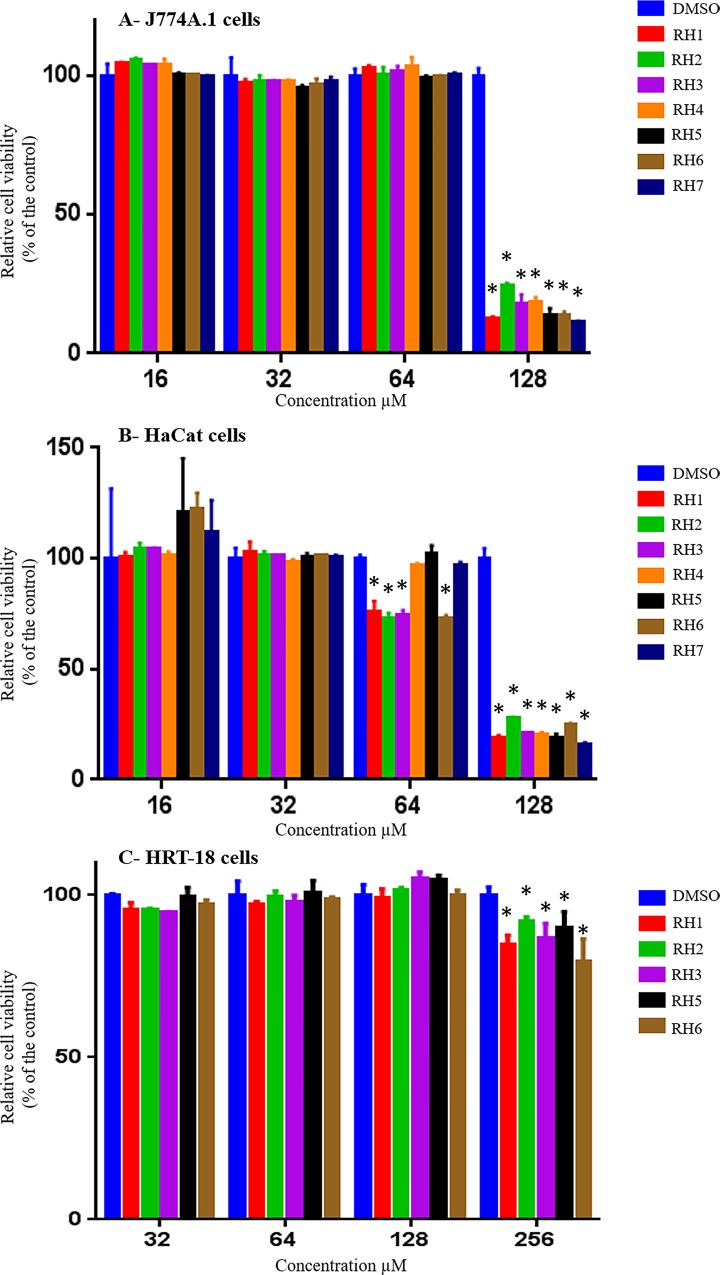
Average absorbance ratio (relative cell viability) for cytotoxicity of rhodanine compounds against murine macrophage cells (J774.A1) (A), human keratinocytes (HaCat) (B), and human ileocecal colorectal (HRT-18) (C), using the MTS 3-(4,5-dimethylthiazol-2-yl)-5-(3-carboxymethoxyphenyl)-2-(4-sulfophenyl)-2*H*-tetrazolium) assay. DMSO was used as a negative control to determine a baseline measurement for the cytotoxic impact of each compound. The absorbance values represent an average of a minimum of three samples analyzed for each compound. Error bars represent standard deviation values for the corrected absorbance values. A paired t-test, P-value ≤ 0.05, demonstrated statistical difference between the values obtained for compounds relative to the cells treated with DMSO.

We determined the cytotoxicity of the rhodanine compounds by using the following mammalian cell lines: murine macrophage (J774A.1), human keratinocyte (HaCat) and human ileocecal colorectal (HRT-18). (Fig 3A, 3B and 3C) The CC_50_s (concentration of drug that results in toxicity to 50% of the cells) of the rhodanine compounds against J774A.1 and HaCat cells were > 64 μM. The CC_50_ against HRT-18 cells for all rhodanine compounds was >256 μM. These results suggest that the tested rhodanine compounds are not cytotoxic to mammalian cells at concentrations significantly higher than the MIC or MBC.

### *In vitro* antibacterial activity of rhodanine compounds 1 to 10 against MRSA USA300 in the presence of human serum albumin

The MIC values of rhodanine compounds **1**–**7** against MRSA USA300 were increased by 8- to 16-fold in the presence of 4% HSA when compared to the MICs obtained in the absence of HSA. Rhodanine **2** ethyl ester (Rh **8**) and amide (Rh **10**) and rhodanine **3** ethyl ester (Rh **9**) were not active in the presence or absence of HSA (Table 11). This finding indicates that the rhodanines bind to HSA and their antibacterial efficacy is subsequently nullified. It also indicates that free carboxylic acid group is essential for antimicrobial activity and that esterification or amidation of the carboxylic acid group abolishes the antibacterial activity *in vitro*.

**Table 11:** Antimicrobial activity of Rhodanine compounds against MRSA USA300 in the presence of human serum albumin.

Media	MIC of Rhodanines (μM) against MRSA USA300											
	Rh 1	Rh 2	Rh 3	Rh 4	Rh 5	Rh 6	Rh 7	Rh 8	Rh 9	Rh 10	Vancomycin	Daptomycin
**TSB**	8	4	8	16	16	8	16	>64	>64	>64	0.5	4
**TSB + 4% HSA**	>64	>64	>64	>64	>64	>64	>64	>64	>64	>64	0.5	64

### The effect of outer membrane and efflux pump of Gram-negative bacteria on rhodanines resistance

Our initial results indicated that the rhodanines did not possess antibacterial activity against Gram-negative bacteria. The lack of efficacy of the rhodanines led us to investigate if the presence of the outer membrane (OM) in Gram-negative bacteria contributed to the lack of antibacterial activity observed, by preventing the rhodanines from gaining entry into the bacterial cell (as has been observed with conventional antimicrobials such as erythromycin and fusidic acid) [13,14]. The inclusion of the permeabilizing agent such as subinhibitory concentration of colistin or polymixin B nonapeptide (PMBN) in the culture broth did not alter the activity of rhodanine against Gram-negative bacteria. (Tables 12 & 13).

**Table 12:** MICs of rhodanines against Gram-negative pathogens in the presence of sub MIC concentration of Colistin.

	MIC of colistin (μg/ml)	Sub-MIC concentration of colistin used (μg/ml)	Rh 1 (μM)		Rh 2 (μM)		Rh 3 (μM)		Rh 4 (μM)		Rh 5 (μM)		Rh 6 (μM)		Rh 7 (μM)		Erythromycin (μM)		Fusidic acid (μM)		Linezolid (μM)		Daptomycin (μM)	
			Colistin		Colistin		colistin		colistin		Colistin		colistin		Colistin		colistin		Colistin		colistin		colistin	
			(-)	(+)	(-)	(+)	(-)	(+)	(-)	(+)	(-)	(+)	(-)	(+)	(-)	(+)	(-)	(+)	(-)	(+)	(-)	(+)	(-)	(+)
***Acinetobacter baumannii* ATCC BAA19606**	0.5	0.0625	>128	>64	>128	>64	>128	>64	>128	>64	>128	>64	>128	>64	>128	>64	16	2	128	≤0.5	>128	>64	>128	>64
***Acinetobacter baumannii* ATCC BAA747**	0.25	0.0625	>128	>64	>128	>64	>128	>64	>128	>64	>128	>64	>128	>64	>128	>64	4	1	>128	1	>128	>64	>128	>64
***Escherichia coli* O157:H7 ATCC 700728**	0.0625	0.0625	>128	>64	>128	>64	>128	>64	>128	>64	>128	>64	>128	>64	>128	>64	32	≤0.5	>128	≤0.5	>128	4	>128	>64
***Escherichia coli* O157:H7 ATCC 35150**	0.125	0.0625	>128	>64	>128	>64	>128	>64	>128	>64	>128	>64	>128	>64	>128	>64	32	≤0.5	>128	≤0.5	>128	≤0.5	>128	>64
***Salmonella Typhimurium* ATCC 700720**	1	0.25	>128	>64	>128	>64	>128	>64	>128	>64	>128	>64	>128	>64	>128	>64	64	16	>128	64	>128	>64	>128	>64
***Klebsiella pneumoniae* ATCC BAA 2146**	0.5	0.125	>128	>64	>128	>64	>128	>64	>128	>64	>128	>64	>128	>64	>128	>64	>128	>64	>128	>64	>128	>64	>128	>64
***Pseudomonas aeruginosa* ATCC 921**	1	0.25	>128	>64	>128	>64	>128	>64	>128	>64	>128	>64	>128	>64	>128	>64	64	16	>128	>64	>128	64	>128	>64

**Table 13:** MICs of Rhodanines against Gram negative pathogens in the presence and absence of polymixin B nonapeptide (PMBN) (4 μg/mL).

Bacterial strain	MIC of PMBN (μg/ml)	Rh 1		Rh 2		Rh 3		Rh 4		Rh 5		Rh 6		Rh 7		Erythromycin		Fusidic acid		Linezolid		Daptomycin	
		(μM)		(μM)		(μM)		(μM)		(μM)		(μM)		(μM)		(μM)		(μM)		(μM)		(μM)	
		PMBN		PMBN		PMBN		PMBN		PMBN		PMBN		PMBN		PMBN		PMBN		PMBN		PMBN	
		(-)	(+)	(-)	(+)	(-)	(+)	(-)	(+)	(-)	(+)	(-)	(+)	(-)	(+)	(-)	(+)	(-)	(+)	(-)	(+)	(-)	(+)
***Acinetobacter baumannii* ATCC BAA19606**	>128	>128	>64	>128	>64	>128	>64	>128	>64	>128	>64	>128	>64	>128	>64	16	2	128	2	>128	>64	>128	>64
***Acinetobacter baumannii* ATCC BAA747**	>128	>128	>64	>128	>64	>128	>64	>128	>64	>128	>64	>128	>64	>128	>64	4	≤0.5	>128	2	>128	>64	>128	>64
***Escherichia coli* O157:H7 ATCC 700728**	>128	>128	>64	>128	>64	>128	>64	>128	>64	>128	>64	>128	>64	>128	>64	32	2	>128	32	>128	128	>128	>64
***Escherichia coli* O157:H7 ATCC 35150**	>128	>256	8	>256	8	>256	8	>256	8	>256	8	>256	8	>256	8	128	4	>256	4	>256	>256	>256	>256
***Salmonella Typhimurium* ATCC 700720**	>128	>128	>128	>128	>128	>128	>128	>128	>128	>128	>128	>128	>128	>128	>128	64	4	>128	64	>128	64	>128	>128
***Klebsiella pneumoniae* ATCC BAA 2146**	>128	>128	>64	>128	>64	>128	>64	>128	>64	>128	>64	>128	>64	>128	>64	>128	>64	>128	>64	>128	>64	>128	>64
***Klebsiella pneumoniae* ATCC BAA 1706**	>128	>128	>64	>128	>64	>128	>64	>128	>64	>128	>64	>128	>64	>128	>64	>128	64	>128	16	>128	>64	>128	>64
***Pseudomonas aeruginosa* ATCC 9721**	>128	>128	>64	>128	>64	>128	>64	>128	>64	>128	>64	>128	>64	>128	>64	64	1	>128	4	>128	16	>128	>64

In addition, we tested the effect of efflux pump AcrAB on the lack of efficacy of the rhodanine compounds in *E*. *coli* using AcrAB defective strain of *E*. *coli*. AcrAB has been shown to contribute to the antibiotic-resistant phenotype in multiple strains of *E*. *coli* and has been implicated in *E*. *coli* resistance to numerous antibiotics including ampicillin, rifampicin, and chloramphenicol [15]. The lack of antimicrobial efficacy of rhodanine compounds in Gram-negative pathogens was not related to the presence of efflux pumps (such as AcrAB) as shown in Table 14. This lack of efficacy against Gram-negative pathogens indicates that these compounds are active only against certain Gram-positive bacteria.

**Table 14:** MICs of Rhodanine and control antibiotics against Escherichia coli Δ acrAB.

*E*. *coli* strain	Rh 1 (μM)	Rh 2 (μM)	Rh 3 (μM)	Rh 4 (μM)	Rh 5 (μM)	Rh 6 (μM)	Rh 7 (μM)	Erythromycin (μM)	Fusidic acid (μM)	Linezolid (μM)	Daptomycin (μM)
***E*.*coli* 1411**	>64	>64	>64	>64	>64	>64	>64	>64	>64	>64	>64
***E*.*coli* 1411 SM**	>64	>64	>64	>64	>64	>64	>64	2	4	16	>64

## Discussion

Bacterial infections account for a substantial proportion of mortality worldwide. Furthermore, the pace of antimicrobial drug discovery to combat these infections has slowed down.

Recently, we synthesized compounds known as rhodanines and determined their efficacy *in vitro* against various MRSA strains [1,3]. Our results indicated that certain rhodanine derivatives were efficacious against six clinically relevant MRSA strains [1]. However, their efficacy against other bacterial strains remained to be determined. The rhodanine compounds characterized in this study had *in vitro* antibacterial efficacy against various strains of VRE, MRSA and VRSA. In addition, the majority of the rhodanine compounds were bactericidal, which is congruent with our previous results for MRSA strains [1]. Overall, rhodanine **2** was the most efficacious compound against the Gram-positive strains tested in this study and this may be attributed to the combined effect of the biaryl ring system substituted with 3,4-dichloro groups. Given the increasing rates of resistance among various multidrug-resistant (MDR) Gram-positive bacterial strains, the rhodanine compounds could offer another treatment modality. In addition, given that the rhodanines are structurally distinct from all currently approved anti-bacterials, it is likely that they would be efficacious against the above tested Gram-positive bacteria in strains resistant to other clinically used drugs.

Bactericidal antibiotics offer many advantages over bacteriostatic antibiotics due to diminished emergence of bacterial resistance to the antibiotics, which in turn can limit the spread of infection [16]. Therefore, rhodanine compounds **1**–**7** were assessed to find out if their inhibition of bacterial growth was bacteriostatic or bactericidal. The majority of these rhodanine compounds were bactericidal as evident from either identical or 2–4 fold higher MBC values compared to their MIC values. This is in contrast to the positive control drug, linezolid, which is predominantly bacteriostatic, and this can pose problems in clearing certain bacterial infections in immune compromised patients and increase the likelihood of drug resistance with prolonged and recurrent infections [17,18].

Rhodanines **1**–**3** had comparable activity against *Mycobacterium smegmatis*, *Bacillus cereus* and *Bacillus anthracis*. Most broad-spectrum antibacterials significantly decrease or eradicate commensal gut microflora. This allows for the colonization of the colon by *C*. *difficile* as an opportunistic bacterium causing colitis. Currently, *C*. *difficile* infections can be treated only with vancomycin, metronidazole or fidaxomicin. In addition, relapse after treatment with vancomycin and metronidazole can occur due to the spore form of *C*. *difficile* [19]. Therefore, new compounds are needed for the treatment of *C*. *difficile* colitis. Rhodanine compounds **1**–**7** may serve as a potential treatment of *C*. *difficile* associated diarrhea. All of the rhodanines were efficacious against all tested strains of *C*. *difficile*, with rhodanines **3**, **4** and **7** being the most potent. However, testing in an *in vivo* model would be required to determine if the rhodanine compounds are safe and efficacious. In addition, the effect of rhodanines on the normal gastrointestinal microflora needs to be determined.

*S*. *epidermidis* is generally a harmless commensal bacterium that is present on skin of all humans. However, under certain conditions, such as implantation of prostheses, *S*. *epidermidis* becomes an invasive species that can produce severe and life-threatening infections. Furthermore, *S*. *epidermidis* produces an abundant and thick biofilm, thereby making significantly less susceptible or even resistant to most antimicrobials [10]. Therefore, we determined the effect of compound **2**, which had the most potent efficacy of all the rhodanines, on the already formed *S*. *epidermidis* biofilms. Rhodanine **2**, significantly reduced biofilm mass by 35% and 45% at 4- and 8-times the MIC, respectively. In contrast, biofilm mass was not significantly decreased at high concentrations of linezolid or vancomycin. These results indicate that rhodanine **2** reduces adherent biofilms produced by *S*. *epidermidis*. This is notable because biofilms can produce protracted infections and increase the likelihood of infection dissemination, drug resistance and mortality. Our results suggest that rhodanine **2** be tested using *in vivo* models of topical *S*. *epidermidis*-related biofilms, and other staphylococcal infections.

The excellent antibacterial profile of rhodanine compounds **1**–**7** prompted us to examine them for potential cytotoxicity against mammalian cells. The cytotoxicity assays were performed to determine whether bacterial cell killing is specific and not a result of general cellular toxicity. At concentrations up to 64 μM (a 16 to 32-fold greater than MIC values), none of these compounds showed significant cytotoxicity against murine macrophage, human keratinocyte and human ileocecal colorectal cell lines.

The rhodanine compounds did not inhibit the growth of the Gram-negative bacteria *P*. *aeruginosa*, *K*. *pneumoniae*, *S*. *typhimurium*, *E*. *coli or Acinetobacter spp*. We sought to investigate if the presence of the outer membrane (OM) and/or the action of efflux pumps in Gram-negative bacteria contributed to the lack of antibacterial activity observed, by preventing rhodanines from gaining entry into the bacterial cell (as has been observed with conventional antimicrobials such as erythromycin and fusidic acid) [13,14]. The inclusion of the permeabilizing agent such as subinhibitory concentration of colistin or PMBN in the culture broth did not alter the activity of rhodanine against Gram-negative bacteria. The lack of susceptibility of the sensitized-Gram negative bacteria to rhodanines suggests either insufficient permeabilization of the OM or that the OM is not a primary barrier to antimicrobial activity for these compounds. We postulated that the rhodanines could be a substrate for an efflux pump (or pumps), thereby decreasing their intracellular levels and thus compromising or eliminating their antibacterial efficacy. However, the rhodanines had no antibacterial efficacy against the *E*. *coli* SM1411Δ acrAB strain, which is deficient in the multidrug-resistant AcrAB efflux pump. Thus, the rhodanines lack of antibacterial activity was not due to its efflux by AcrAB. However, it is possible that the rhodanines could be substrate for other efflux pumps, although this remains to be determined.

Previously, it has been reported that certain rhodanine compounds have antifungal activity[20]. Consequently, we determined the efficacy of the rhodanine derivatives against 10 strains of *C*. *albicans*. Our results indicated that amphotericin B, a wide spectrum antifungal drug, significantly inhibited the growth of all of the *C*. *albican*s strains. However, none of the rhodanines in this study was efficacious against *C*. *albicans*.

In this study, compound **2** was identified as a lead compound as it showed excellent growth inhibition of a wide range of Gram-positive bacteria. In addition, its toxicity occurred at much higher concentrations than the MIC. However, compound **2** and the other six compounds showed a significant shift in MIC in the presence of HSA, which may be a consequence of the high lipophilicity and acidic nature of these compounds as mentioned before. Therefore, compound **2** is not suitable for *in vivo* antibacterial evaluation. Hence, we sought to modify carboxylic acid group to ester and amide (Rh **8**–**10**) in order to reduce human serum protein binding. However, these variations in the chemical structure proved to be detrimental to the antibacterial activity (Table 11). Therefore, we will initiate structural modifications of compound **2** to decrease its binding to HSA and increase its antibacterial potency. These goals can be achieved by reducing the lipophilicity. Potency enhancement can be achieved by core structure modifications such as cyclopropanation of the benzylidene C = C bond at the C5 of the rhodanine core. This is anticipated to increase the three dimensionality of the molecule, which in turn will decrease lipophilicity[21]. Moreover, this molecular configuration will reveal its role in enhancing the antibacterial activity profile. We will replace biphenyl moiety with various biaryl systems, where one or both phenyl rings would be replaced with heteroaromatic rings. Saturated heterocycles can also be installed instead of the aromatic ring system. We will also make cell—penetrating isosteres of the carboxyl group such as tetrazole.

In conclusion, the rhodanine compounds, particularly **2**, were active *in vitro* against a number of MDR Gram-positive cocci, *C*. *difficile*, *Bacillus spp*., and *M*. *smegmatis*. Future studies include synthesizing and testing derivatives of rhodanine **2** to increase potency and minimize protein binding.

## Supporting Information

S1 Table Bacterial isolates used in Rhodanines study and synthesis of ester and amide derivatives. (DOCX)
