# Supplementary material for: In Vitro Antibacterial Activity of Rhodanine Derivatives against Pathogenic Clinical Isolates
Source: PLoS One. 2016 Oct 6;11(10):e0164227. doi: 10.1371/journal.pone.0164227 (PMC5053523; doi:10.1371/journal.pone.0164227)
Supplement: S1 Table — (DOCX) [file pone.0164227.s001.docx]

**Table 1: Bacterial isolates used in Rhodanines study**

| **Name of Bacterial strains** | **Strain ID** | **Source** | **Description** |
| --- | --- | --- | --- |
| **Vancomycin-resistant *Staphylococcus aureus* (VRSA)** | | | |
| Vancomycin-resistant *Staphylococcus aureus* (VRSA) | HIP11714, VRSA-1, VRSA-MI | Isolated in 2002 in Michigan, USA from an infected catheter exit site of a 40-year-old adult who had a co-infection with vancomycin-resistant Enterococcus faecalis (E. faecalis) and had been treated with numerous antibiotics, including a six-week course of vancomycin therapy. | The first documented VRSA strain in the USA. Resistant to vancomycin as well as number of other antibiotics, it is positive for *mec* (subtype II) and *vanA*; negative for *vanB*, *vanC1*, *vanC2*, *vanD*, *vanE*, PVL and arginine catabolic mobile element (ACME); pulsed-field type USA100; MLST (ST) 5; spa repeats TJMGMK; Ridom spa type t062. |
| Vancomycin-resistant *Staphylococcus aureus* (VRSA) | HIP11983, VRSA-2 | Isolated in 2002 in Pennsylvania, USA, from a plantar ulcer of a 70-year-old male with cellulitis along the plantar fascia and polymicrobial osteomyelitis of the right calcaneus and who had no recent history of vancomycin therapy. | This strain is vancomycin resistant, positive for *mec* (subtype II) and *vanA*; negative for *vanB*, *vanC1*, *vanC2*, *vanD*, *vanE*, PVL and arginine catabolic mobile element (ACME) genes; pulsed-field type USA100; spa repeats TJMBMDMGMK; Ridom spa type t002. |
| Vancomycin-resistant *Staphylococcus aureus* (VRSA) | HIP13170, VRSA-3a | Isolated in 2004 in New York, USA, from urine of a 63-year-old female with a polymicrobial infected nephrostomy tube exit site who had no recent history of vancomycin therapy. | This strain was isolated from the third documented case of VRSA infection in the United States. Positive for *mec* (subtype IV), *vanA* and resistance genes against tetracycline, macrolides, lincosamides and aminoglycosides; negative for *vanB*, *vanC1*, *vanC2*, *vanD* and *vanE*; pulsed-field type USA800; MLST (ST) 5; spa repeats TJMBMDMGMK; Ridom spa type t002. It was co-isolated with S. aureus, strain HIP13419 (VRS3b) which are similar in most aspects except the vancomycin resistant phenotype for HIP13170 is less stable than that of HIP13419. |
| Vancomycin-resistant *Staphylococcus aureus* (VRSA) | HIP13419, VRSA3b | Isolated in 2004 in New York, USA, from a polymicrobial infected nephrostomy tube exit site of a 64- year-old female who had no recent history of vancomycin therapy. | It is positive for *mec* (subtype IV), *vanA* and resistance genes against tetracycline, macrolides, lincosamides and aminoglycosides; negative for *vanB*, *vanC1*, *vanC2*, *vanD* and *vanE*; pulsed-field type USA800; MLST (ST) 5; spa repeats TJMBMDMGMK; Ridom spa type t002. Strains HIP13170 and HIP13419 were isolated from the third documented case of VRSA infection in the United states and are believed to be identical. Because of this, characterization has primarily been performed for strain HIP13170 (VRS3a). |
| Vancomycin-resistant *Staphylococcus aureus* (VRSA) | HIP14300, VRSA-4 | Isolated in 2005 in Michigan, USA from a gangrenous toe wound of a 78-year-old male nursing home resident who was recently treated with a nine-week course of vancomycin. | It is positive for *mec* (subtype II) and *vanA*; negative for *vanB*, *vanC1*, *vanC2*, *vanD*, *vanE*, PVL and arginine catabolic mobile element (ACME); pulsed-field type USA100; spa repeats TJMBMDMGMK; Ridom spa type t002.1 |
| Vancomycin-resistant *Staphylococcus aureus* (VRSA) | HIP15178, VRSA-5 | Isolated in 2005 in Michigan, USA from a surgical site infection of a 58-year-old female who was recently treated with an eight-week course of vancomycin. | This strain is positive for *mec* (subtype II) and *vanA*; negative for *vanB*, *vanC1*, *vanC2*, *vanD*, *vanE*, PVL and arginine catabolic mobile element (ACME); pulsed-field type USA100; spa repeats TJMBMDMGMK; Ridom spa type t002. |
| Vancomycin-resistant *Staphylococcus aureus* (VRSA) | AIS2006032, VRSA6 | Isolated in 2005 in Michigan, USA, from infected non-healing plantar ulcers of a 48-year-old male who had a history of long term vancomycin therapy. | It is positive for *vanA*; negative for *vanB*, *vanC1*, *vanC2*, *vanD*, *vanE*, PVL and arginine catabolic mobile element (ACME); pulsed-field type USA100; spa repeats TJMGMK; Ridom spa type t062.1 |
| Vancomycin-resistant *Staphylococcus aureus* (VRSA) | AIS 2006045, VRSA-7 | Isolated in 2006 in Michigan, USA from a triceps wound of a 43-year-old female with necrotizing fasciitis of the right upper limb who had been recently treated with a five-week course of vancomycin. | It is positive for *mec* (subtype II) and *vanA*; negative for *vanB*, *vanC1*, *vanC2*, *vanD*, *vanE*, PVL and arginine catabolic mobile element (ACME); pulsed-field type USA100; spa repeats TJMBMDMGMK; Ridom spa type t002.1 |
| Vancomycin-resistant *Staphylococcus aureus* (VRSA) | 71080, VRSA-8 | Isolated in 2007 in Michigan, USA from a toe wound of a 48-year-old female who had recently received a 7-month course of vancomycin and ceftriaxone to treat osteomyelitis of the right metatarsals. | The strain is positive for *mec* and *vanA*; negative for *vanB*, *vanC1*, *vanC2*, *vanD*, *vanE*, PVL and arginine catabolic mobile element (ACME); pulsed-field type USA100; spa repeats TJMBMDMGMK; Ridom spa type t002. |
| Vancomycin-resistant *Staphylococcus aureus* (VRSA) | AIS 080003, VRSA-9 | Isolated in 2007 in Michigan, USA from a left plantar foot wound of a 54-year-old female who recently received a 4-week course of vancomycin and levofloxacin to treat osteomyelitis of the left metatarsals | It is positive for *mec* and *vanA*; negative for *vanB*, *vanC1*, *vanC2*, *vanD*, *vanE*, PVL and arginine catabolic mobile element (ACME); pulsed-field type USA100; spa repeats TJMBMDMGMK; Ridom spa type t002. S. aureus, strain AIS080003 is partially dependent on vancomycin for growth due to a mutation in D-alanyl:D-alanine ligase (Ddl), an enzyme involved in biosynthesis of eptidoglycans. The presence of the *vanA* operon compensates for the Ddl mutation by providing alternative peptidoglycans for cell wall synthesis; however, some have found that this change in the type of peptidoglycans produced renders it susceptible to oxacillin. |
| Vancomycin-resistant *Staphylococcus aureus* (VRSA) | AIS 1000505, VRSA-10 | Isolated in 2009 from a plantar foot wound of a 53-year-old female in Michigan, USA. | Positive for *mec* and *vanA*; negative for *vanB*, PVL and arginine catabolic mobile element (ACME); pulsed-field type USA100; spa repeats TJMBMDMGMK; Ridom spa type t002. |
| Vancomycin-resistant *Staphylococcus aureus* (VRSA) | AIS 1001095, VRSA-11a | Isolated in 2010 in Delaware, USA from wound drainage of a 63-year-old female with a prosthetic joint infection who had been unsuccessfully treated with continuous vancomycin therapy for 3 months. | It is positive for *mec* and *vanA*; negative for *vanB*, PVL and arginine catabolic mobile element (ACME); pulsed-field type USA100; spa repeats TJMBMDMGMK; Ridom spa type t002. Strain AIS 1001095 is a co-isolate with strain AID 1001123 (VRS11b) from the same patient and they were isolated at the same time. Both are *mecA* positive but unlike strain AID 1001123, strain AIS 1001095 is phenotypically susceptible to oxacillin by the cefoxitin disk diffusion test. |
| Vancomycin-resistant *Staphylococcus aureus* (VRSA) | AID 1001123, VRSA-11b | Isolated in 2010 in Delaware, USA from wound drainage of a 63-year-old female with a prosthetic joint infection who had been unsuccessfully treated with continuous vancomycin therapy for 3 months. | Positive for mecA and vanA; negative for vanB, PVL and arginine catabolic mobile element (ACME); pulsed-field type USA100; spa repeats TJMBMDMGMK; Ridom spa type t002. |
| ***Bacillus* Strains** | | | |
| *Bacillus cereus* NR-22150 | VD148 | Isolated in 2008 from a soil sample collected in Scotland, United Kingdom. | It is reported to contain a pXO2-like plasmid1-3 and is part of a Bacillus cereus Database Sequencing Project at the Broad Institute. |
| *Bacillus licheniformis* NR-2499 | NRS 712 | Isolated in 1938 from flour | It was deposited at ATCC® in 1945 by Dr. Nathan R. Smith. This strain reportedly produces D-glutamic acid polypeptide. |
| *Bacillus licheniformis* NR-2494 | Gibson 46 (NCIB 9375, DSM 13) | Isolated by Dr. T. Gibson | It was deposited at ATCC® in 1962 by Dr. Ruth E. Gordon, Dr. Ruth E. Gordon, Institute of Microbiology, Rutgers University, New Brunswick, New Jersey. |
| *Bacillus cereus* NR-22148 | VD115 | Isolated in 2008 from a soil sample collected in Guadeloupe, French West Indies | It is reported to contain a pXO2-like plasmid1-3 and is part of a Bacillus cereus Database Sequencing Project at the Broad Institute. |
| *Bacillus cereus* NR-28575 | BAG1X1-1 | Bacillus cereus (B. cereus), strain BAG1X1- 1 was isolated in 2009 from a soil sample collected in Boston, Massachusetts, USA | It is reported to contain a pXO1-like plasmid1 and is part of a Bacillus cereus Database Sequencing Project at the Broad Institute |
| *Bacillus cereus* NR-28582 | BAG1O-2 | It was isolated in 2009 from a soil sample collected in Boston, Massachusetts, USA | It is part of a Bacillus cereus Database Sequencing Project at the Broad Institute. |
| *Bacillus cereus* NR-28578 | BAG1X2-1 | Isolated in 2009 from a soil sample collected in Boston, Massachusetts, USA | It is reported to contain a pXO2-like plasmid1 and is part of a Bacillus cereus Database Sequencing Project at the Broad Institute |
| *Bacillus cereus* NR-2488 | NRS 201 | Isolated from blood by L. Siribaed | It was deposited at ATCC® by Dr. N. R. Smith as Bacillus siamensis. 2 This strain reportedly has enterotoxin activity3 and does not produce zwittermicin A. |
| *Bacillus cereus* NR-9564 | G9241 | Isolated from sputum and blood of a welder with life-threatening pneumonia in Louisiana, 1994 | It contains 2 large plasmids known as pBCXO1 and pBC218. pBCXO1 has significant homology to B. anthracis pXO1 and harbors the entire anthrax toxin biosynthetic complex. pBC218 contains genes capable of capsule production, however they are not homologous to the B. anthracis capsule genes found on pXO2.5 B. cereus, strain G9241 contains genes that may provide resistance to β-lactam, chloramphenicol, and macrolide antibiotics. |
| *Bacillus cereus* NR-22141 | VD014 | Isolated in 2008 from a soil sample collected in Andalusia, Spain. | It is reported to contain a pXO1-like plasmid1 and is part of a Bacillus cereus Database Sequencing Project at the Broad Institute. |
| *Bacillus anthracis*  NR-10355 | Ames35 (ANR-1) | It is a derivative of the Ames strain that was treated with novobiocin to cure it of the pXO2 plasmid. | The parent strain of strain Ames35 was isolated from a dead cow in Jim Hogg County, Texas in 1981 and was fully virulent, containing both pXO1 and pXO2 plasmids. |
| *Bacillus anthracis*  NR-10351 | UM23 | It is a Ura− derivative of the Weybridge strain. | The presence of pX01 and absence of pX02 in NR-10351 has been confirmed by PCR amplification of plasmid-specific sequences from extracted DNA. |
| *Bacillus anthracis*  NR-10350 | Weybridge | It was isolated by the Microbiological Research Establishment (MRE) in Porton Down, England (MRE closed in 1979). | Strain Weybridge is an avirulent strain that contains the toxigenic pXO1 plasmid but lacks the pXO2 capsule plasmid. |
| **Vancomycin Resistant *Enterococci* (VRE) Strains** | | | |
| *E. faecalis* HM-335 | R712,  HMP ID 9377 | Isolated in 2004 from the blood of a 64-year-old female hemodialysis patient with fatal bacteremia. It was from blood drawn after treatment with daptomycin | Reported to be resistant to daptomycin. This strain is a reference genome for The Human Microbiome Project (HMP). HMP is an initiative to identify and characterize human microbial flora. |
| *E. faecalis* HM-934 | ERV103,  HMP ID 1328 | This strain is a clinical isolate from a human secretion in Bogota, Colombia, in 2006 | Reference genome for The Human Microbiome Project (HMP). |
| *E. faecalis* HM-334 | S613,  HMP ID 9376 | Isolated in 2004 from the blood of a 64-year-old female hemodialysis patient with fatal bacteremia. The S613 isolate was from blood drawn before treatment with daptomycin.1 | Reported to be susceptible to daptomycin. This strain is a reference genome for The Human Microbiome Project (HMP). |
| *E. faecalis* HM-201 | TX0104,  HMP ID 348 | Isolated in January, 2002 from the blood of an endocarditis patient at Stamford Hospital in Connecticut, USA | Reported to be vancomycin resistant. This strain is a reference genome for The Human Microbiome Project (HMP). |
| *E. faecalis* NR31972 | SF 28073 | Isolated in 2003 from a human urine sample obtained in Michigan, USA | It is reported to be resistant to erythromycin, gentamicin and vancomycin |
| *E. Faecium* NR31914 | E0120 (EnGen0012) | Isolated in 1995 from ascites fluid of a hospitalized patient in the Netherlands | Reported to be resistant to gentamicin and vancomycin. |
| *E. faecium* NR-31903 | Patient #1-1 (EnGen0314) | Isolated from the stool of a human patient prior to bacteremia. | The complete genome of E. faecium, strain Patient #1-1 (EnGen0314) has been sequenced. |
| *E. faecium* HM-965 | E417 | Isolated from human blood in Ecuador, 2006 | Reported to be resistant to ampicillin and vancomycin, and displays high levels of resistance to gentamycin and streptomycin. It is a reference genome for The Human Microbiome Project (HMP). |
| *E. faecium* NR-28978 | E1071 | Collected from the feces of a hospitalized person free of enterococcal infection in the Netherlands in 2000 during a hospital surveillance program | It is a non-infectious fecal isolate |
| *E. Faecium* HM 968 | ERV102,  HMP ID 1362 | Isolated from human oral sputum collected in Colombia, 2006 | Reported to be resistant to ampicillin and vancomycin, and displays high levels of resistance to streptomycin. It is a reference genome for The Human Microbiome Project (HMP). |
| ***Mycobacterium*** | | | |
| *M. Smegmatis* ATCC 14468 | W-113 [113; ATCC 101; 178, NCIB 9953, NCTC 10265, TMC 1546] | Deposited Name: Mycobacterium smegmatis (Trevisan) Lehmann and Neumann |  |
|  | | | |
| ***Staphylococcus* strains** | | | |
| *Staphylococcus aureus* NRS 382 | 626 (USA100 and New York/Japan clone) | Isolated from a bloodstream sample in Ohio, USA | It is a methicillin-resistant S. aureus (MRSA) strain. It was deposited as resistant to erythromycin, clindamycin and levofloxacin; positive for *mec* (subtype II); sed+ ; pulsed-field type USA100; MLST sequence type (ST) 5; eGenomic spa type 2, eGenomic spa repeats TJMBMDMGMK; Ridom spa type t002; agr grp II.1 It is a USA100 isolate.. USA100 is the most prevalent U.S health care-associated pulse-field type and is endemic in many U.S. hospitals |
| *Staphylococcus aureus* NRS 383 | 96758 (also referred to as USA200) | Isolated from a bloodstream sample in North Carolina, USA. 1 | Methicillin-resistant S. aureus (MRSA) strain. It was deposited as resistant to erythromycin, clindamycin and gentamicin; positive for *mec* (subtype II), *tsst* and *sea*; pulsed-field type (PFT) USA200; MLST sequence type (ST) 36; eGenomic spa type 16, eGenomic spa repeats WGKAKAOMQQQ; Ridom spa type t018; agr grp III.1,2 S. aureus, strain 96758 is a USA200 isolate. USA200 is the second most common health care associated pulsed-field type in U.S. |
| *Staphylococcus aureus* NRS 384 | USA300-0114 | Isolated from a wound in Mississippi, USA. | It is a community acquired methicillin-resistant S. aureus (CA-MRSA) strain. Outbreaks of this strain have been reported in correctional facilities (located in MS, GA, TN, TX, and CA); athletic teams (located in PA and CA); and male homosexual behavior (observed in CA). Strain USA300-0114 was deposited as resistant to erythromycin and tetracycline; positive for *mec* (subtype IV); pvl+ ; MLST sequence type (ST) 8; eGenomic spa type 1, eGenomic spa repeats YHGFMBQBLO; Ridom spa type t008; agr grp I. Note: Methicillin is no longer clinically used, however, the term methicillin-resistant Staphylococcus aureus (MRSA) continues to be used to describe S. aureus strains resistant to all penicillins |
| *Staphylococcus aureus* NRS 385 | 95938 (USA500) | Isolated from a bloodstream sample in Connecticut, USA. | S. aureus, strain 95938 is a hospital-acquired methicillin-resistant S. aureus (HA-MRSA) strain. It was deposited as resistant to erythromycin, clindamycin, trimethoprim/sulfamethoxazole, levofloxacin, gentamicin and tetracycline; positive for *mec* (subtype IV), *sea* and *seb*; MLST sequence type (ST) 8; pulsed-field type USA500; eGenomic spa type 7, eGenomic spa repeats YHGCMBQBLO; Ridom spa type t064; agr grp I. It is a USA500 isolate. USA500 is believed to be the predecessor of the most common community-associated pulsed-field type, USA300. |
| *Staphylococcus aureus* NRS 386 | 1078 (USA700) | Isolated from a bloodstream sample in Louisiana, USA | It is a methicillin-resistant S. aureus (MRSA) strain. S. aureus, strain 1078 was deposited as resistant to erythromycin; positive for *mec* (subtype IV); MLST sequence type (ST) 72; pulsed-field type USA700; eGenomic spa type 49, eGenomic spa repeats UJGFMGGM; Ridom spa type t126; agr grp I. S. aureus, strain 1078 is a USA700 isolate. USA700 is associated with infections in both community and healthcare settings. |
| *Staphylococcus aureus* NRS387 | 1045 (USA800) | Isolated from a wound in Washington, USA | Methicillin-resistant S. aureus (MRSA) strain. It was deposited as positive for *mec* (subtype IV) and *seb*; pulsedfield type (PFT) USA800; MLST sequence type (ST) 5; eGenomic spa type 29, eGenomic spa repeats TJMBMDMGGMK; Ridom spa type t088; agr grp II. It is a USA800/Pediatric isolate. USA 800 isolates are resistant to β- lactams and fluoroquinolones with some isolates being resistant to additional antibiotics. While first isolated in pediatric patients, USA800 strains recently have been isolated in adults. |
| *Staphylococcus aureus* NRS 107 | RN4220/pG0400 (G0400) | Isolated in 1991 during an outbreak of **mupirocin**-**resistant** S. aureus on a dermatology ward of a university hospital in Connecticut, USA | It contains the plasmid pG0400 and is a transconjugant of mating between S. aureus, strain G03221 containing the plasmid pG0400 and S. aureus, strain RN4220NR. Plasmid pG0400 is a 33.8 kilobase plasmid that encodes **resistance to mupirocin.** S. aureus, strain RN4220/pG0400 is a methicillin-sensitive S. aureus (MSSA) strain. It was deposited as containing the plasmid pG0400; resistant to mupirocin, rifampicin and novobiocin; negative for *mec*; MLST sequence type (ST) 8; eGenomic spa type 59, eGenomic spa repeats YHGGFMBQBLO; Ridom spa type t211. |
| *Staphylococcus aureus* NRS 1 | Mu50 | Isolated from pus and debrided tissue at surgical incision in sternum of 4 month-old infant, Japan, 1996. | This strain shows reduced vancomycin susceptibility (**vancomycin intermediately** resistant starain). |
| *Staphylococcus aureus* NRS 19 | HIP07256 | Isolated in 1999 in Illinois, USA from the bloodstream of a 67-year-old male ICU patient with vertebral osteomyelitis that was secondary to recurrent methicillin-resistant S. aureus (MRSA) cellulitis and bacteremia and who had a prior 14-week course of vancomycin therapy | It is a **vancomycin intermediate** S. aureus (**VISA**) strain. It was deposited as positive for *mec* (subtype II); negative for *vanA*, *vanB*, *vanC1*, *vanC2*, *vanD*, and *vanE*; MLST sequencing type (ST) 5; eGenomic spa type 2, eGenomic spa repeats TJMBMDMGMK; Ridom spa type t002 |
| *Staphylococcus aureus* NRS 37 | LIM 3 | Isolated in 1995 from purulent discharge of a 2- year-old female patient with leukemia in France. 1 | Methicillin-resistant S. aureus (MRSA) and a **vancomycin-intermediate** S. aureus (VISA) strain. S. aureus, strain LIM 3 was deposited as positive for *mec* (subtype I); negative for *vanA*, *vanB*, *vanC1*, *vanC2*, *vanD*, and *vanE*; MLST sequence type (ST) 247, eGenomic spa type 4, eGenomic spa repeats YHFGFMBQBLO; Ridom spa type t051.1 Strain LIM 3 was the third MRSA isolate recovered from this patient. During the course of treatment, which included the use of glycopeptides, three additional MRSA strains, LIM 1 (NRS35), LIM 2 (NRS36) and LIM 4, were isolated. LIM 1 was the first strain isolated from the patient and has a vancomycin-sensitive S. aureus (VSSA) phenotype whereas LIM 2 and LIM 4 have a VISA phenotype. |
| *Staphylococcus aureus* NRS 119 | SA LinR #12 | Isolated in 2001 from an 85-year-old male with dialysis-associated peritonitis in Massachusetts, USA. | Methicillin resistant S. aureus (MRSA) strain.1 It was deposited as **resistant to linezolid**; positive for *mec* (subtype IV); MLST sequence type (ST) 507; eGenomic spa type 7, eGenomic spa repeats YHGCMBQBLO; Ridom spa type t064. It was co-isolated with SA LinR #13 (NRS120) and SA LinR #14 (NRS121) from the first clinically reported case of a MRSA infection that demonstrated resistance to linezolid. Based on pulsed-field gel electrophoresis, SA LinR #12 and SA LinR #13 are identical and SA LinR #14 is closely related to both. While each strain has a different antibiogram, all three are resistant to linezolid due to a G2576T mutation in domain V in one or more 23S rRNA genes (Escherichia coli numbering). Since this initial case, additional linezolid resistant S. aureus (LRSA) strains have been isolated, including ones that have the same G2576T point mutation in their 23S rRNA genes |
| ***Candida* Strains** | | | |
| *C. albicans* NR 29435 | P57072 | Candida albicans (C. albicans), strain P57072 is a bloodstream isolate from a person with a bloodstream infection collected in Iowa City, Iowa, USA, in 2000 | Strain P57072 is a member of genetic clade II and has an alpha/alpha MTL genotype. |
| *C. albicans* ATCC 10231 | 3147 | Isolated from man with bronchomycosis | This strain is recommended by ATCC for use in the tests described in ASTM Standard Test Method E979-91 where only the taxon is specified. Assay of amphotericin B fungizone, Assay of antimicrobial preservatives, Assay of haloprogin, Assay of nystatin fungicidin and Media testing |
| *C. albicans* NR 29436 | P34048 | Isolate from the blood of a person with an infection collected in Istanbul, Turkey, in 2001. | Strain P34048 has an alpha/alpha MTL genotype |
| *C. albicans* NR 29449 | 19F | It is a vaginal isolate from a person with vaginitis collected in Ann Arbor, Michigan, USA, between 1990 and 1992. | Strain 19F is a member of genetic clade I2 and is known to have an α/α MTL genotype |
| *C. albicans* NR29438 | P75016 | Isolate from the blood of a person with an infection, collected in Tel-Hashomer, Israel, in 2000 | Strain P75016 has an a/alpha MTL genotype |
| *C. albicans* NR 29434 | P78048 | Isolate from the blood of a person with an infection collected in Winnipeg, Manitoba, Canada, in 2000 | Strain P78048 is a member of genetic clade I and has an alpha/alpha MTL genotype |
| *C. albicans* NR29437 | P75010 | Candida albicans (C. albicans), strain P75010 is a bloodstream isolate from a person with a bloodstream infection collected in Brussels, Belgium in 2000 | Strain P75010 is a member of genetic clade E and has an a/alpha MTL genotype. |
| *C. albicans* NR 29453 | P87 | Candida albicans (C. albicans), strain P87 is an oral isolate from an HIV+ person collected in Pretoria, South Africa | Strain P87 is a member of genetic clade SA2 and has an a/a MTL genotype |
| *C. albicans* NR 29448 | P60002 | Candida albicans (C. albicans), strain P60002 is an isolate from a person with a bloodstream infection, collected in Arizona, USA | Strain P60002 is known to have an a/a MTL genotype |
| *C. albicans* NR 29446 | P94015 | Candida albicans (C. albicans), strain P94015 is a bloodstream isolate from a person with a bloodstream infection collected in Utah, USA. | Strain P94015 is a member of genetic clade I and has an a/a MTL genotype. |
| **Gram negative strains** | | | |
| *Acinetobacter baumannii* ATCC BAA19606 | 2208 [81, DSM 6974] | isolated from urine sample | Used as quality control strain and in media testing |
| *Acinetobacter baumannii,* | Naval-81, NR-17786 | Isolated on October 9, 2006, from human blood at the National Naval Medical Center in Bethesda, MD, USA | It is part of the "Genomic Sequencing of a Diversity of US Military*Acinetobacter baumannii-calcoaceticus* Complex Isolates" project to sequence the genomes of clinical and environmental isolates of medically elevant *Acinetobacter* spp. |
| *Acinetobacter baumannii* | NR-13375 Isolate 2 | Isolated from human sputum in 2008, | One of the major causes of nosocomial infectionsit acquires antibiotic resistance rapidly due to the presence of resistance islands that can carry up to 45 resistance genes. |
| *Escherichia coli* O157:H7 ATCC 700728 | BDMS T4169 [LMG 21756, NCTC 12900] |  | Quality control strain for BBL chromagar |
| *Escherichia coli* O157:H7 ATCC 35150 | EDL 931 | Isolated from human feces. | Presence of *eaeA*, *stx1*, and *stx2* genes confirmed by PCR, Cytotoxic activity, Hemorrhagic colitis, Growth on Novobiocin (10 mg/L), Growth on Tellurite (0.8 mg/L) |
| *Salmonella Typhimurium* ATCC 700720 | LT2 | Unknown -- wild type strain isolated from a natural source, 1948 | Used in Emerging infectious disease research |
| *Klebsiella pneumoniae* | NR-15412, Isolate 3 |  | Multi-drug resistant K. pneumoniae isolate 3 contains the β-lactamase K. pneumoniae carbapenemase (blaKPC) gene. The presence of the blaKPC gene has been confirmed by PCR amplification of a blaKPC gene specific sequence from extracted DNA. Resistant to all antibiotics except gentamicin and tetracycline. |
| *Klebsiella pneumoniae* | NR-15471, Isolate 8 |  | Multi-drug resistant K. pneumoniae isolate 8 contains the β-lactamase K. pneumoniae carbapenemase (blaKPC) gene. The presence of the blaKPC gene has been confirmed by PCR amplification of a blaKPC gene specific sequence from extracted DNA. Resistant to all antibiotics except amikacin and tetracycline. |
| *Klebsiella pneumoniae* ATCC BAA 2146 | 1000527, 7561 | Isolated from human Urine | New Delhi metallo-beta-lactamase (NDM-1) positive, *blaKPC* negative by PCR, blaNDM positive by PCR. Used for Multidrug testing and respiratory research |
| *Klebsiella pneumoniae* ATCC BAA 1706 | AIS 2007023 [6179 | --- | *blaKPC* negative by PCR |
| *Pseudomonas aeruginosa* | ATCC 15442, PRD-10 | Isolated from the environment, | *Quality control strain* |
| *Pseudomonas aeruginosa* | ATCC 9721 |  | *Quality control strain* |
| *Pseudomonas aeruginosa* ATCC 9721 | NRS 112 [NRRL B-7, R. Hugh 814] |  | Used for bacterial resistance testing adhesives, Produces lipases active at pH 5.5 and 7.5 |
| ***Clostridium* strains** | | | |
| *Clostridium difficile*  HM-746 | Strain 002-P50-2011 | Isolated in January 2011 from the stool of a patient with diarrhea. | It is a reference genome for The Human Microbiome Project (HMP). |
| *Clostridium difficile*  HM-88 | Strain NAP07 (CDC#2007054) | Isolated from human feces | It is a reference genome for The Human Microbiome Project (HMP). |
| *Clostridium difficile*  NR-13427 | Isolate 1 | It was obtained from a human patient from the Mid-Atlantic region of the United States in 2008/2009. | - |
| *Clostridium difficile*  NR-32888 | Strain P8 | It was obtained in 2001 from fecal material of a human patient with a C. difficile infection in western Pennsylvania, USA. | Strain P8 is deposited as a toxigenic strain. |
| *Clostridium difficile*  HM-745 | Strain 70-100-2010 | It was isolated in June 2010 from the stool of a patient with diarrhea. | It is a reference genome for The Human Microbiome Project (HMP). |

**Experimental**

**General**

The reagents for organic synthesis were purchased from Sigma Aldrich (St. Louis, MO), TCI America (Portland, OR), Alfa Aesar (Ward Hill, MA), Avantor (Center Valley, PA) and Acros Organics (Antwerp, Belgium) and were used as received. All compounds were evaluated for homogeneity by TLC using silica gel as a stationary phase. NMR spectra were recorded on a Bruker 400 Avance DPX spectrometer (^1^H at 400 MHz) outfitted with a z-axis gradient probe. The chemical shifts for ^1^H are reported in parts per million (δ ppm) downfield from tetramethylsilane (TMS), which was used as an internal standard. The ^1^H NMR data are reported as follows: chemical shift, multiplicity (s) singlet, (d) doublet, (t) triplet, and (m) multiplet, respectively. HPLC analysis was performed using Agilent 1100 Series instrument. The compounds were dissolved in acetonitrile and injected (10 µL) into the C18 column (Agilent Eclipse plus C18; 3.5 µm; 4.6 x 100 mm). The elutions were obtained using an isocratic mobile phase (90:10 acetonitrile/water) at a flow rate of 1 mL/min. The elutions were monitored at UV 370 nm. The retention times (*t*_R_) are given in minutes.

**Synthesis of ester and amide derivatives**

**Ethyl (*L*,*Z*)-2-(5-((3',4'-dichloro-[1,1'-biphenyl]-3-yl)methylene)-4-oxo-2-thioxothiazolidin-3-yl)-3-phenylpropanoate (8)**

Compound **8** was synthesized by a modified procedure^1^. A solution of acid (**2**, 100 mg, 0.19 mmol) in anhydrous ethanol (15 mL) was added to *p*-toluenesulfonic acid monohydrate (148 mg, 0.77 mmol) and heated to reflux for 24 h. Afterwards, excess amount of *p*-toluenesulfonic acid monohydrate (148 mg, 0.77 mmol) was added to the refluxing solution and stirred for an additional 48 h. The reaction was cooled once TLC showed that most of the starting acid was no longer present. Ethanol was evaporated and the residue was purified by reverse phase (C_18_) flash chromatography (acetonitrile:water; gradient 0:100 to 100:0) to obtain ester **8** (70 mg, Yield 67%) as yellow oil; R*_f_* = 0.80 (*n*-hexane:ethyl acetate 70:30); ^1^H NMR (400 MHz, CDCl_3_, TMS) δ 7.70 (1H, s), 7. 65 (1H, d, *J =* 2 Hz), 7.61-7.53 (4H, m), 7.48-7.47 (1H, m), 7.42 (1H, d, *J* = 2.4 Hz), 7.27-7.21 (5H, m), 5.95 (1H, m), 4.34-4.27 (2H, m), 3.66 (2H, d, *J* = 8 Hz), 1.32 (3H, t, *J* = 7.2 Hz). HPLC *t*_R_ = 9.0 min, purity 94%.

**Ethyl (*L*,*Z*)-2-(5-(3-(3,4-dichlorobenzyl)benzylidene)-4-oxo-2-thioxothiazolidin-3-yl)-3-phenylpropanoate (9)**

The synthesis of compound **9** starting from acid **3** (106 mg, 0.19 mmol) was achieved as per the procedure for compound **8,** with the exception of an additional reaction time of 24 h. Yellow oil (65 mg, Yield 58%); R*_f_* = 0.80 (*n*-hexane:ethyl acetate 70:30); ^1^H NMR (400 MHz, CDCl_3_, TMS) δ 7.64 (1H, s), 7.46-7.35 (4H, m), 7.29-7.20 (7H, m), 7.04-7.03 (1H, m), 5.94-5.93 (1H, m), 4.31-4.28 (2H, m), 3.99 (2H, s), 3.65 (2H, d, *J* = 7.6 Hz), 1.30 (3H, t, *J* = 7.2 Hz). HPLC *t*_R_ = 7.4 min, purity 95%.

(***L***,***Z*)-2-(5-((3',4'-dichloro-[1,1'-biphenyl]-3-yl)methylene)-4-oxo-2-thioxothiazolidin-3-yl)-3-phenylpropanamide (10)**

Amide **10** was prepared from acid (**2**) following the reported procedure^1^ with minor variations. Briefly, compound **2** (100 mg, 0.19 mmol) was dissolved in *N*,*N*-dimethyl formamide (5 mL). Subsequently, the following reagents were added sequentially: *N*-hydroxybenzotriazole (60 mg, 0.39 mmol), ammonium chloride (21 mg, 0.39 mmol), *N*-methylmorpholine (0.1 mL, 0.77 mmol) and 1-(3- dimethylaminopropyl)-3-ethylcarbodiimide hydrochloride (75 mg, 0.39 mmol). The reaction mixture was then stirred for 48 h at rt after which it was diluted with ethyl acetate and washed with 3N HCl (1 X 30 mL), followed by brine (1 X 30 mL). The organic extract was dried over magnesium sulfate and concentrated *in vacuo,* leaving a yellow crude liquid that was purified using flash chromatography to give compound **10** (50 mg, Yield 50%) as a yellow solid; R*_f_* = 0.20 (*n*-hexane:ethyl acetate 70:30); ^1^H NMR (400 MHz, DMSO-d_6_, TMS) δ 8.02 (2H, d, *J* = 9.6 Hz), 7. 87 (1H, d, *J =* 8 Hz), 7.82-7.76 (3H, m), 7.69-7.64 (2H, m), 7.58 (1H, d, *J* = 7.2 Hz), 7.40 (1H, s), 7.22-7.18 (2H, m), 7.15-7.12 (3H, m), 5.72 (1H, s), 3.57-3.56 (2H, m). HPLC *t*_R_ = 2.8 min, purity 97%.

1. Orchard MG, Neuss JC, Galley CM, inventors; Oxford Glycosciences (Uk) Ltd., assignee. Benzylidene thiazolidinediones and their use as antimycotic agents. United States patent US 7,105,554. 2006 Sep 12.





Scheme 1: Synthesis of an ester and amide derivatives of rhodanines **2** and **3**
